# After the War, What?

**Published:** 1917-05

**Authors:** 


### After the War, What?

The medical profession will attain a new dignity and importance following this great world-wide war. It is demonstrating the value of medical knowledge and surgical skill as a modern factor in war in such a way as to make an impression upon the world that will redound to the credit of the profession. There was a time that war killed more men from disease than were killed in battle, when medical skill seemed totally unable to cope with the usual conditions of war, but now health conditions are almost as good along battle lines as along the streets of our best kept cities. A man badly wounded was then given over to die, but now as long as there is life there is hope, and a good prospect, too, that the wounded can be patched up and spared to serve his country with much usefulness.

However, medical men are being lost in the war faster than they are being graduated in the colleges. Most of the European schools have been closed for some time, and even after the war the student ranks of that country will be so depleted that the professional schools will be almost empty. Of course, should the war continue, many of our students will soon be on the battle grounds, but there will be thousands left at home who will for various reasons be unfitted for military service. These young men will find that the practice of medicine will be an especially attractive field of useful labor both in this and in foreign countries when the world-wide war is over, for medicine and surgery will have a position even higher than it has ever held, and there will be fewer prepared for the practice.

Another result of the war is sure to be a large increase in the number of women who will study medicine and the various branches of surgery. More and more women are becoming medical practitioners, and the Red Cross and surgical service of the war will take from surgery many of the horrors it has held for women practitioners. The professions have, and with some justice, rather reluctantly been opened to women, due in a large measure to their lack of that patient application requisite to professional success, but the lessons of the war are already showing them that professional success along any line can only be attained by the hardest study and through a serious determination to succeed.

There is no danger that there will be a crowding of the professions of medicine and surgery for many decades after the war, and for this reason the medical schools of this country, unless prevented by war exigencies that cannot now be foreseen, should not abate in the least their efforts to supply the country with capable physicians and surgeons. Until the war in Europe, it seemed that the medical colleges were turning out too many graduates, but the war has changed conditions and created new demands.

